# Effects of pharmacological treatment on metabolomic alterations in animal models of depression

**DOI:** 10.1038/s41398-022-01947-5

**Published:** 2022-04-29

**Authors:** Juncai Pu, Yiyun Liu, Siwen Gui, Lu Tian, Yue Yu, Dongfang Wang, Xiaogang Zhong, Weiyi Chen, Xiaopeng Chen, Yue Chen, Xiang Chen, Xue Gong, Lanxiang Liu, Wenxia Li, Haiyang Wang, Peng Xie

**Affiliations:** 1 Department of Neurology, The First Affiliated Hospital of Chongqing Medical University, Chongqing, 400016 China; 2 NHC Key Laboratory of Diagnosis and Treatment on Brain Functional Diseases, The First Affiliated Hospital of Chongqing Medical University, Chongqing, 400016 China; 3 Department of Health Sciences Research, Mayo Clinic, MN 55901 USA

**Keywords:** Depression, Prognostic markers

## Abstract

Numerous studies have investigated metabolite alterations resulting from pharmacological treatment in depression models although few quantitative studies explored metabolites exhibiting constant alterations. This study aimed to identify consistently dysregulated metabolites across such studies using a knowledgebase-driven approach. This study was based on 157 studies that identified an assembly of 2757 differential metabolites in the brain, blood, urine, liver, and feces samples of depression models with pharmacological medication. The use of a vote-counting approach to identify consistently upregulated and downregulated metabolites showed that serotonin, dopamine, norepinephrine, gamma-aminobutyric acid, anandamide, tryptophan, hypoxanthine, and 3-methoxytyramine were upregulated in the brain, while quinolinic acid, glutamic acid, 5-hydroxyindoleacetic acid, myo-inositol, lactic acid, and the kynurenine/tryptophan ratio were downregulated. Circulating levels of trimethylamine *N*-oxide, isoleucine, leucine, tryptophan, creatine, serotonin, valine, betaine, and low-density lipoprotein were elevated. In contrast, levels of alpha-d-glucose, lactic acid, *N*-acetyl glycoprotein, glutamine, beta-d-glucose, corticosterone, alanine, phenylacetylglycine, glycine, high-density lipoprotein, arachidonic acid, myo-inositol, allantoin, and taurine were decreased. Moreover, 12 metabolites in urine and nine metabolites in the liver were dysregulated after treatment. Pharmacological treatment also increased fecal levels of butyric acid, acetic acid, propionic acid, and isovaleric acid. Collectively, metabolite disturbances induced by depression were reversed by pharmacological treatment. Pharmacological medication reversed the reduction of brain neurotransmitters caused by depression, modulated disturbance of the tryptophan-kynurenine pathway and inflammatory activation, and alleviated abnormalities of amino acid metabolism, energy metabolism, lipid metabolism, and gut microbiota-derived metabolites.

## Introduction

Depression is a common mental illness characterized by low mood, diminished interest, and a reduced sense of pleasure, with an incidence of 6.4 to 10.4% [1, 2]. Depression severely reduces the quality of life of patients and decreases their life expectancy by ~10 years [3]. The global burden of disease due to depression has been increasing in recent decades and depression has become one of the leading causes of disability worldwide [4]. In 2018, the economic burden of depression in the United States alone was US$326.2 billion [5]. Despite the significant harm caused by depression, much uncertainty still exists about the complete molecular mechanism of depression, making it a major challenge to identify new antidepressants [6].

Classical antidepressants exert antidepressant properties by modulating monoaminergic targets. A recent meta-analysis concluded that all licensed antidepressants have a significant therapeutic effect in adults with acute depression [7]. Extensive research showing antidepressant properties of pharmacological treatments other than monoaminergic antidepressants, such as ketamine, adjunctive nutraceuticals, Traditional Chinese Medicine, and probiotics, has progressively attracted interest [8–12]. However, the underlying therapeutic mechanisms of classical antidepressants and other pharmacological treatments are not fully understood [13–15].

Metabolomics has been widely used to quantify metabolites in an organism or tissue and identify relationships between metabolites and physiological or pathological processes [16]. Indeed, because of its ability to elucidate metabolic alterations induced by treatments, metabolomics has become a vital tool for drug discovery [17]. With the merit of high accuracy in detecting the content of small molecules associated with depression (such as neurotransmitters, amino acids, lipids, and energy metabolites), metabolomic methods have emerged as powerful tools for exploring molecular alterations induced by pharmacological treatments in the brain or other tissues in animal models of depression [18–20]. Moreover, because metabolites are easily absorbed and can penetrate the blood–brain barrier, they are an important source for screening potential drug leads for the treatment of depression [21, 22].

Several issues have been raised for metabolomics studies investigating potential molecular mechanisms of antidepressant action. First, although extensive studies have been carried out, it remains difficult to detect panoramic alterations of metabolites induced by pharmacological treatment in individual studies; accordingly, a systematic understanding of the neurochemical effects of these medications is still lacking. Second, the generalizability of much-published research on this issue is problematic, as experimental data are rather controversial due to small sample sizes and differences in experimental design. Faced with challenges surrounding how to systematically analyze these massive metabolomics data and a lack of accessible quantitative data [23, 24], few studies have explored the reliability and reproducibility of findings for metabolites across metabolomics studies.

Thus, the purpose of this study was to investigate metabolite changes consistently resulting from pharmacological treatment in depression models using a knowledgebase-driven approach. To this end, a vote-counting method was used to identify consistently upregulated and downregulated metabolites in the brain and peripheral tissues of animal models induced by pharmacological treatment from large-scale metabolomics studies.

## Materials and methods

### Data source

The dataset used in this study was obtained from the MENDA database. Details of the study design and procedures for data collection and annotation are described elsewhere [25]. In brief, after screening more than 11,000 citations from literature and metabolomics databases as of March 2018, we collected relevant information from 464 studies that investigated metabolite alterations associated with depression and antidepressant treatment in human and animal models. Based on metabolomics and magnetic resonance spectroscopy techniques, a total of 5675 differential metabolites or metabolite ratios were identified.

As a result of the current study, we updated the MENDA database as follows. After screening more than 18,000 citations as of 11 August 2021, we cumulatively retrieved 2278 potentially eligible articles and then excluded 1461 full-text studies. Excluded studies are listed in Supplementary Data 1. In total, 817 clinical and preclinical studies were included in the MENDA database. From these studies, an assembly of 13,112 differential metabolite entries was manually curated.

### Candidate metabolite set

For further study, we selected the candidate metabolite set according to the following steps. Only studies that aimed to identify differential metabolites resulting from pharmacological treatment in animal models of depression were included. Therefore, we excluded studies that (i) compared metabolite levels between depressed and control conditions; (ii) focused on non-pharmacological treatments, such as repetitive transcranial magnetic stimulation, electroacupuncture, or exercise therapy; (iii) explored metabolic changes after pharmacological treatment in a healthy state; or (iv) investigated metabolic characteristics associated with treatment response. Human and nonhuman primate models were also excluded. This decision was made a priori because metabolic profiles are clearly different between rodents, humans, and nonhuman primates [26]. We focused on rodent models because there was a large amount of data on brain tissues provided by rodent samples, while few studies have examined metabolomic changes in the brain tissues of human or nonhuman primates. For analytical techniques, untargeted or targeted metabolomics studies that used mass spectrometry or nuclear magnetic resonance methods were included, while in vivo studies employing magnetic resonance spectroscopy techniques were excluded. Candidate metabolites in brain, blood, urine, liver, and feces samples were selected for analysis, whereas other tissues were excluded due to a limited amount of data.

### Analytic strategy

In this study, we used the vote-counting method to identify metabolites consistently upregulated or downregulated in the candidate metabolite set. Although combining effect sizes has been the mainstream method for integrating multiple clinical metabolomics studies [27–29], meta-analyses cannot be performed because of the significant loss of quantitative data (mean concentrations and standard deviation) in the evaluated animal studies. Alternatively, the advantage of the vote-counting method is that it avoids problems associated with a lack of statistical data. Currently, it is the most feasible method for merging such a large number of studies because differential metabolites were described in most studies. Moreover, vote counting is an effective method for selecting molecules to be independently validated by external studies, with the assumption that a reliable differential molecule is robust enough to show significance across multiple studies [30]. This approach has been recently implemented in other research integrating large-scale metabolomics studies [31, 32].

In the current study, the following analyses were performed based on the vote-counting procedure. In the primary analysis, we analyzed metabolite alterations in brain, blood, urine, liver, and feces samples of animal models resulting from all available pharmacological treatments. Next, secondary analyses were performed based on tissue types and pharmacological treatments. For the types of tissues, we analyzed candidate metabolites in the hippocampus, prefrontal cortex, hypothalamus, plasma, and serum samples. For the types of pharmacological treatments, eight licensed drugs for depression (citalopram, fluoxetine, paroxetine, venlafaxine, amitriptyline, desipramine, imipramine, and vortioxetine) were classified as antidepressants [7], and pharmacological treatments other than these licensed antidepressants (various types of compounds and Traditional Chinese Medicine) were classified as non-antidepressants. In addition, probiotics and fecal microbiota transplantation were classified as non-antidepressant therapy because they yield antidepressant effects by ameliorating metabolomic disturbances and modifying the colonic ecosystem [33].

### Statistical analysis

The vote-counting method is based on the hypothesis that the probability of upregulation or downregulation of a differential metabolite follows a binomial distribution, i.e., there is a random occurrence of significant upregulation or downregulation for the altered metabolite in each study. In this study, we calculated the vote-counting statistic (VCS) for each metabolite in the vote-counting procedure. Each metabolite entry exhibiting an upregulation or downregulation trend was scored with 1 or −1 points, respectively. Next, the VCS for each metabolite was calculated by computing the total score of all studies, with VCS values more than 0 indicating that the candidate metabolite was predisposed to be upregulated across studies, and vice versa. Duplicate metabolites curated from the same study but with different study designs (including different types of drugs, dose groups, types of tissues, and analytical techniques) were considered to be derived from independent studies. For each metabolite, the one-tailed *P* value of the binomial test was estimated to compare hypothesized versus obtained counts of upregulation and downregulation trends, using the *binom.test* function in R version 4.0.4 (https://www.R-project.org/). A *P* value < 0.05 was considered statistically significant. Metabolites with total counts of dysregulation less than four were not introduced into the vote-counting procedures because their minimum *P* values were 0.125.

## Results

### Data sets

After data selection, we excluded 660 studies from the MENDA database (listed in Supplementary Data 2). At the metabolite level, we excluded 10,355 differential metabolite entries from the 13,112 metabolite entries. The reasons for these excluded studies and metabolite entries are summarized in Supplementary Table 1. Detailed information of the 157 studies and 2757 metabolite entries used in the current study are presented in Supplementary Data 3 and Supplementary Data 4, respectively. Next, metabolic alterations in the brain, blood, urine, liver, and feces were investigated by vote-counting procedures based on the candidate metabolite set. The numbers of studies and metabolite entries for each tissue are summarized in Supplementary Table 2.

### Effects of pharmacological treatment on metabolite alterations in the brain

**Fig. 1:**
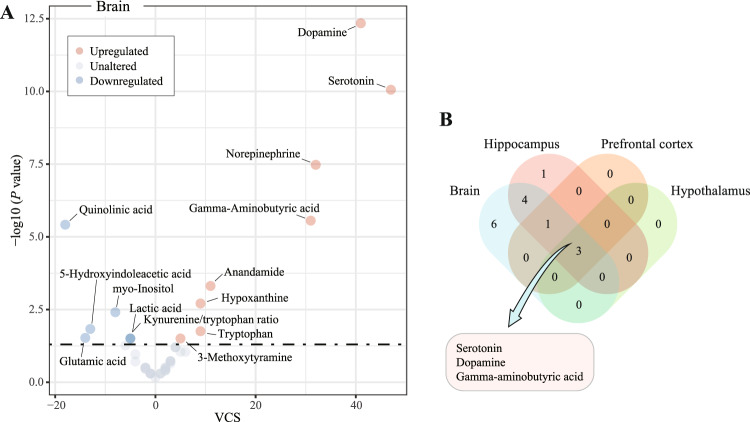
Effects of pharmacological treatment on metabolite alterations in the brain of depression models. **A** Volcano plot for consistently upregulated (red) and downregulated (blue) metabolites across studies. Dysregulated metabolites were identified by vote-counting procedures with a significance threshold of one-tailed *P* < 0.05. The vote-counting statistic (VCS) is presented on the *x*-axis and the −log10 (*p* value) is presented on the *y*-axis. **B** Venn plot for shared dysregulated metabolites between the brain, hippocampus, prefrontal cortex, and hypothalamus.

Among 46 metabolites and one metabolite ratio that were voted in the brain, eight and six were consistently upregulated and downregulated, respectively (Fig. 1A). Specifically, serotonin (VCS = 47, *P* < 0.001), dopamine (VCS = 41, *P* < 0.001), norepinephrine (VCS = 32, *P* < 0.001), gamma-aminobutyric acid (GABA, VCS = 31, *P* < 0.001), anandamide (VCS = 11, *P* < 0.001), hypoxanthine (VCS = 9, *P* = 0.002), tryptophan (VCS = 9, *P* = 0.018), and 3-methoxytyramine (VCS = 5, *P* = 0.031) were upregulated, while quinolinic acid (VCS = −18, *P* < 0.001), glutamic acid (VCS = −14, *P* = 0.030), 5-hydroxyindoleacetic acid (VCS = −13, *P* = 0.015), myo-inositol (VCS = −8, *P* = 0.004), lactic acid (VCS = −5, *P* = 0.031), and the kynurenine/tryptophan ratio (VCS = −5, *P* = 0.031) were downregulated (Supplementary Table 3).

We further analyzed metabolite alterations in the hippocampus (504 metabolite entries), prefrontal cortex (164 metabolite entries), and hypothalamus (39 metabolite entries). Of the 28 candidate metabolites in the hippocampus, six were upregulated and three were downregulated. Specifically, concentrations of serotonin (VCS = 23, *P* < 0.001), GABA (VCS = 17, *P* < 0.001), norepinephrine (VCS = 13, *P* < 0.001), dopamine (VCS = 12, *P* < 0.001), glycine (VCS = 7, *P* = 0.008), and hypoxanthine (VCS = 7, *P* = 0.008) were increased, whereas concentrations of glutamic acid (VCS = −9, *P* = 0.047), myo-inositol (VCS = −8, *P* = 0.004), and lactic acid (VCS = −5, *P* = 0.031) were decreased (Supplementary Table 4). Among the eight candidate metabolites examined in the prefrontal cortex, serotonin (VCS = 12, *P* < 0.001), norepinephrine (VCS = 10, *P* = 0.001), GABA (VCS = 7, *P* = 0.020), and dopamine (VCS = 6, *P* = 0.016) were upregulated (Supplementary Table 5). Among the six candidate metabolites evaluated in the hypothalamus, serotonin (VCS = 5, *P* = 0.031), dopamine (VCS = 5, *P* = 0.031), and GABA (VCS = 5, *P* = 0.031) were upregulated (Supplementary Table 6). Numbers of shared dysregulated metabolites in the hippocampus, prefrontal cortex, and hypothalamus are shown in Fig. 1B. Only serotonin, dopamine, and GABA were shared metabolites in these three brain regions.

### Effects of pharmacological treatment on metabolite alterations in blood

**Fig. 2:**
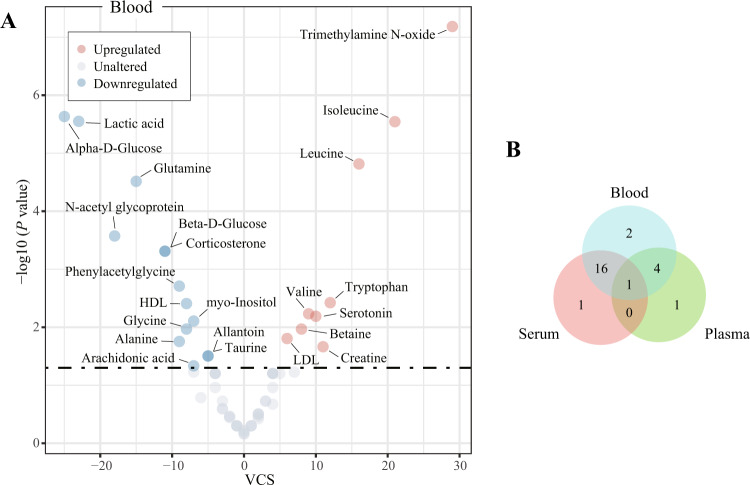
Effects of pharmacological treatment on metabolite alterations in the blood of depression models. **A** Volcano plot for consistently upregulated (red) and downregulated (blue) metabolites across studies. The vote-counting statistic (VCS) is presented on the *x*-axis and the −log10 (*p* value) is presented on the *y*-axis. **B** Venn plot for shared dysregulated metabolites between blood, serum, and plasma. HDL high-density lipoprotein, LDL low-density lipoprotein.

The results of vote counting showed that 23 of the 63 circulating metabolites were consistently dysregulated (Fig. 2A). Specifically, levels of trimethylamine *N*-oxide (VCS = 29, *P* < 0.001), isoleucine (VCS = 21, *P* < 0.001), leucine (VCS = 16, *P* < 0.001), tryptophan (VCS = 12, *P* = 0.004), creatine (VCS = 11, *P* = 0.022), serotonin (VCS = 10, *P* = 0.006), valine (VCS = 9, *P* = 0.006), betaine (VCS = 8, *P* = 0.011), and low-density lipoprotein (LDL; VCS = 6, *P* = 0.016) were upregulated, while levels of alpha-d-glucose (VCS = −25, *P* < 0.001), lactic acid (VCS = −23, *P* < 0.001), *N*-acetyl glycoprotein (VCS = −18, *P* < 0.001), glutamine (VCS = −15, *P* < 0.001), beta-d-glucose (VCS = −11, *P* < 0.001), corticosterone (VCS = −11, *P* < 0.001), alanine (VCS = −9, *P* = 0.018), phenylacetylglycine (VCS = −9, *P* = 0.002), glycine (VCS = −8, *P* = 0.011), high-density lipoprotein (HDL;VCS = −8, *P* = 0.004), arachidonic acid (VCS = −7, *P* = 0.046), myo-inositol (VCS = −7, *P* = 0.008), allantoin (VCS = −5, *P* = 0.031), and taurine (VCS = −5, *P* = 0.031) were downregulated (Supplementary Table 7).

We further analyzed metabolite alterations in plasma (344 metabolite entries) and serum samples (663 metabolite entries). Among the 29 candidate metabolites in plasma, six were dysregulated. Specifically, the concentration of tryptophan (VCS = 10, *P* = 0.001) was increased, and concentrations of corticosterone (VCS = −11, *P* < 0.001), arachidonic acid (VCS = −7, *P* = 0.008), glycine (VCS = −6, *P* = 0.035), d-glucose (VCS = −5, *P* = 0.031), and glutamine (VCS = −5, *P* = 0.031) were decreased (Supplementary Table 8). Eighteen of the 43 candidate metabolites in serum were dysregulated. Specifically, trimethylamine *N*-oxide (VCS = 28, *P* < 0.001), isoleucine (VCS = 21, *P* < 0.001), creatine (VCS = 13, *P* = 0.002), leucine (VCS = 13, *P* < 0.001), pyruvic acid (VCS = 10, *P* = 0.001), betaine (VCS = 7, *P* = 0.020), valine (VCS = 7, *P* = 0.008), and serotonin (VCS = 7, *P* = 0.008) were upregulated, and *N*-acetyl glycoprotein (VCS = −22, *P* < 0.001), alpha-d-glucose (VCS = −21, *P* < 0.001), lactic acid (VCS = −21, *P* < 0.001), glutamine (VCS = −10, *P* = 0.001), HDL (VCS = −8, *P* = 0.004), alanine (VCS = −8, *P* = 0.029), beta-d-glucose (VCS = −7, *P* = 0.008), allantoin (VCS = −5, *P* = 0.031), phenylacetylglycine (VCS = −5, *P* = 0.031), and taurine (VCS = −5, *P* = 0.031) were downregulated (Supplementary Table 9). Numbers of shared dysregulated metabolites in plasma and serum are shown in Fig. 2B, and glutamine was the only shared metabolite.

### Effects of pharmacological treatment on metabolite alterations in urine

**Fig. 3:**
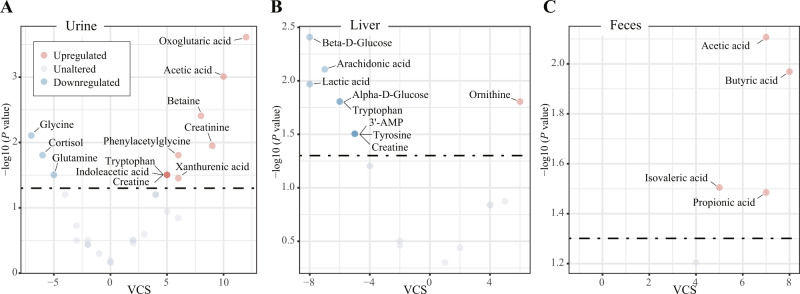
Effects of pharmacological treatment on metabolite alterations in urine, liver, and feces of depression models. Volcano plot for consistently upregulated (red) and downregulated (blue) metabolites in urine (**A**), liver (**B**), and feces (**C**). The vote-counting statistic (VCS) is presented on the *x*-axis and the −log10 (*p* value) is presented on the *y*-axis. AMP adenosine monophosphate.

The results of vote counting showed that 12 of the 33 candidate metabolites in urine were dysregulated (Fig. 3A). Specifically, levels of oxoglutaric acid (VCS = 12, *P* < 0.001), acetic acid (VCS = 10, *P* = 0.001), creatinine (VCS = 9, *P* = 0.011), betaine (VCS = 8, *P* = 0.004), phenylacetylglycine (VCS = 6, *P* = 0.016), xanthurenic acid (VCS = 6, *P* = 0.035), creatine (VCS = 5, *P* = 0.031), indoleacetic acid (VCS = 5, *P* = 0.031), and tryptophan (VCS = 5, *P* = 0.031) were increased, while levels of glycine (VCS = −7, *P* = 0.008), cortisol (VCS = −6, *P* = 0.016), and glutamine (VCS = −5, *P* = 0.031) were decreased (Supplementary Table 10).

### Effects of pharmacological treatment on metabolite alterations in the liver

Of the 18 candidate metabolites that were voted in the liver, one was consistently upregulated and eight were consistently downregulated (Fig. 3B). Specifically, the concentration of ornithine (VCS = 6, *P* = 0.016) was increased, while concentrations of beta-d-glucose (VCS = −8, *P* = 0.004), lactic acid (VCS = −8, *P* = 0.011), arachidonic acid (VCS = −7, *P* = 0.008), alpha-d-glucose (VCS = −6, *P* = 0.016), tryptophan (VCS = −6, *P* = 0.016), 3′-AMP (VCS = −5, *P* = 0.031), creatine (VCS = −5, *P* = 0.031), and tyrosine (VCS = −5, *P* = 0.031) were decreased (Supplementary Table 11).

### Effects of pharmacological treatment on metabolite alterations in feces

Of the five candidate metabolites in feces, four were consistently upregulated, including butyric acid (VCS = 8, *P* = 0.011), acetic acid (VCS = 7, *P* = 0.008), propionic acid (VCS = 7, *P* = 0.033), and isovaleric acid (VCS = 5, *P* = 0.031) (Fig. 3C and Supplementary Table 12).

### Pharmacological treatment reversed metabolite disturbances induced by depression

**Fig. 4:**
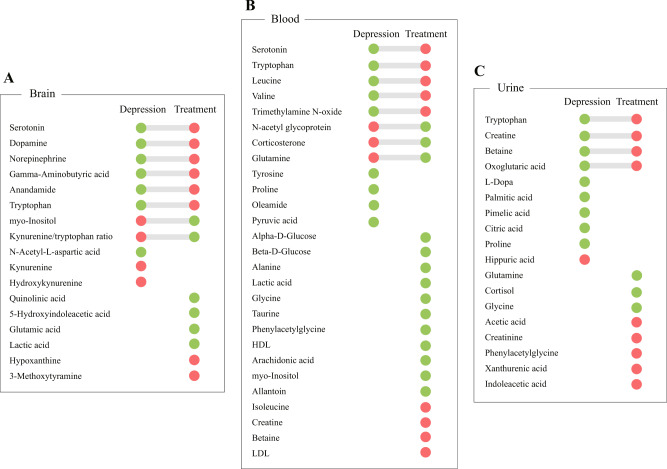
Pharmacological treatment reversed metabolite disturbances induced by depression. Dysregulated metabolites in the brain (**A**), blood (**B**), and urine (**C**) resulting from both depression and pharmacological treatment. Red and green circles in the diagram indicate significant upregulation and downregulation, respectively. For each tissue, circles on the left represent dysregulated metabolites resulting from depression and the right circles represent altered metabolites caused by pharmacological treatment.

To further explore which metabolite disturbances induced by depression can be reversed by pharmacological treatment, we compared the current data with our previous data showing dysregulated metabolites in the brain, blood, and urine samples of depression models [31]. An overview of consistently dysregulated metabolites resulting from depression and pharmacological treatment in animal models is shown in Fig. 4.

Among the 11 dysregulated metabolites or metabolite ratios in the brains of depression models, eight were reversed by pharmacological treatment (Fig. 4A). Specifically, decreased levels of serotonin, dopamine, norepinephrine, GABA, anandamide, and tryptophan, as well as increased levels of myo-inositol and the kynurenine/tryptophan ratio resulting from depression were reversed following treatment. However, increased levels of kynurenine and hydroxykynurenine and the decreased level of *N*-acetyl-L-aspartic acid were not attenuated following treatment.

For metabolite disturbances in blood, pharmacological treatment reversed eight of the 12 dysregulated metabolites, including decreased concentrations of serotonin, tryptophan, leucine, valine, and trimethylamine *N*-oxide, as well as increased concentrations of *N*-acetyl glycoprotein, corticosterone, and glutamine (Fig. 4B). Decreased concentrations of tyrosine, proline, oleamide, and pyruvic acid were not reversed following treatment.

Among the ten dysregulated metabolites resulting from depression in urine, only four downregulated metabolites (tryptophan, creatine, betaine, and oxoglutaric acid) were reversed by pharmacological treatment (Fig. 4C). Dysregulated levels of l-DOPA, palmitic acid, pimelic acid, citric acid, proline, and hippuric acid were not attenuated following treatment.

### Effects of antidepressants and non-antidepressants on metabolite alterations

**Fig. 5:**
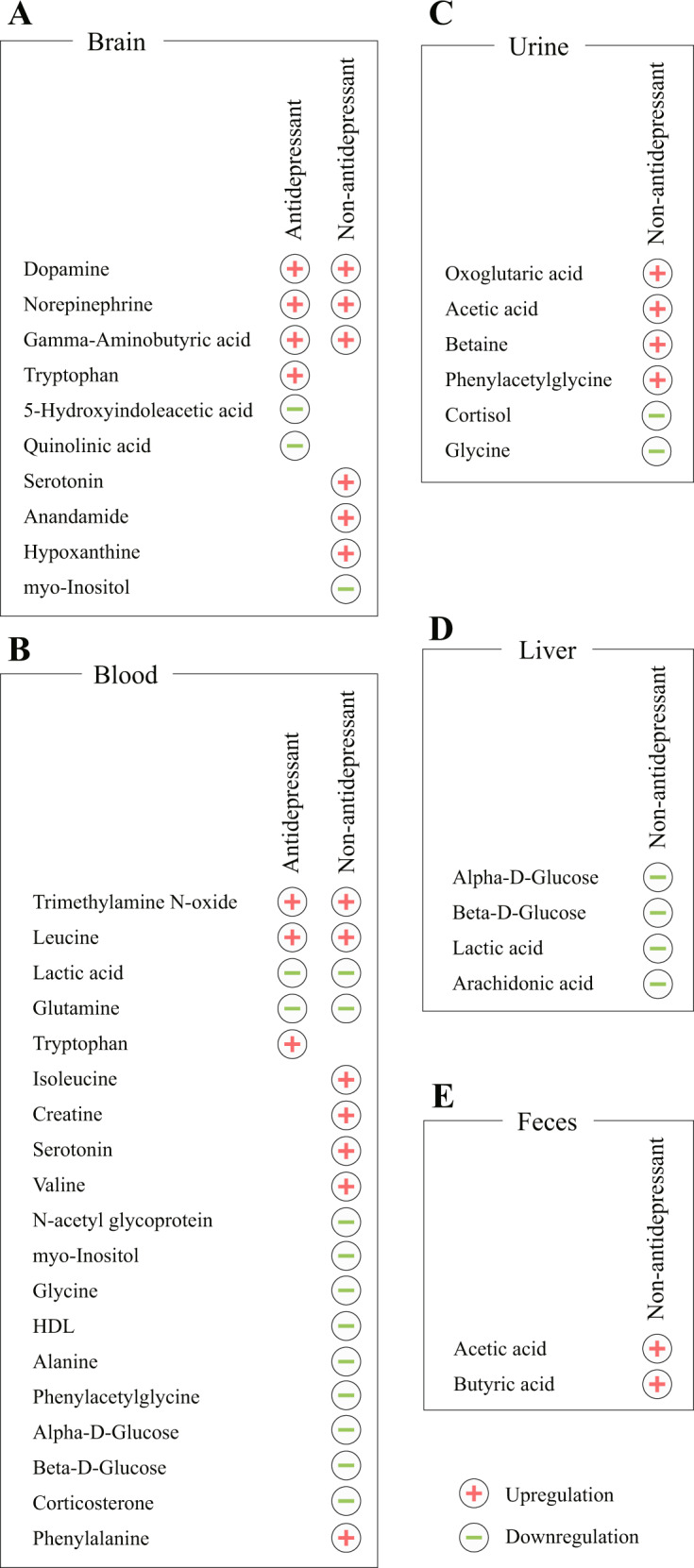
Effects of antidepressants and non-antidepressants on metabolite alterations. Metabolite changes resulting from antidepressants and non-antidepressants in the brain (**A**), blood (**B**), urine (**C**), liver (**D**), and feces (**E**). Red plus and green minus signs in the diagram indicate significant upregulation and downregulation, respectively.

We further explored the commonalities and differences of metabolic effects resulting from antidepressants and non-antidepressants. For metabolites in the brain, a total of 344 and 555 metabolite entries resulting from antidepressants and non-antidepressants were voted, respectively. Of the 15 candidate metabolites of antidepressants, six were dysregulated (Supplementary Table 13). For non-antidepressants, seven of the 26 candidate metabolites were dysregulated (Supplementary Table 14). These altered metabolites are summarized in Fig. 5A. Notably, levels of dopamine, norepinephrine, and GABA were upregulated by both antidepressants and non-antidepressants. In addition, concentrations of tryptophan, 5-hydroxyindoleacetic acid, and quinolinic acid were altered by antidepressants, while concentrations of serotonin, anandamide, hypoxanthine, and myo-inositol were altered by non-antidepressants.

For metabolites in blood, a total of 289 and 718 metabolite entries assessing antidepressants and non-antidepressants were voted, respectively. Among 17 candidate metabolites assessing antidepressants, five were dysregulated (Supplementary Table 15). For non-antidepressants, 18 of the 48 candidate metabolites were dysregulated (Supplementary Table 16). Levels of trimethylamine *N*-oxide and leucine were upregulated by both antidepressants and non-antidepressants, while lactic acid and glutamine were downregulated (Fig. 5B). The concentration of tryptophan was altered by antidepressants, while concentrations of isoleucine, creatine, serotonin, valine, *N*-acetyl glycoprotein, myo-inositol, glycine, HDL, alanine, phenylacetylglycine, alpha-d-glucose, beta-d-glucose, and corticosterone were altered by non-antidepressants.

We next investigated metabolite alterations in urine, liver, and feces. Neither of the two candidate metabolites potentially altered by antidepressants in urine were significantly dysregulated (Supplementary Table 17). Moreover, no candidate metabolites in the liver or feces were voted due to limited numbers of metabolite entries. For non-antidepressants, six candidate metabolites (oxoglutaric acid, acetic acid, betaine, phenylacetylglycine, cortisol, and glycine) were dysregulated in urine (Fig. 5C and Supplementary Table 18), four candidate metabolites (alpha-d-glucose, beta-d-glucose, lactic acid, and arachidonic acid) were decreased in the liver (Fig. 5D and Supplementary Table 19), and two candidate metabolites (acetic acid and butyric acid) were increased in feces (Fig. 5E and Supplementary Table 20).

## Discussion

Although numerous studies have assessed the effects of pharmacological treatment on metabolite levels in depression models, inconsistent results for dysregulation of metabolites were reported. Here, we performed the first systematic investigation to identify consistently dysregulated metabolites using a knowledgebase-driven approach. The results of vote-counting procedures showed that 13 metabolites and one metabolite ratio in the brain, 23 metabolites in blood, 12 metabolites in urine, nine metabolites in the liver, and four metabolites in feces were consistently dysregulated across the evaluated large-scale studies. These findings represent the panoramic metabolomic alterations induced by pharmacological treatment in depression, thus contributing to an understanding of the molecular mechanisms underlying antidepressant effects.

Consistent with our expectations, we confirmed that the regulatory effect of neurotransmitters is the most pronounced feature of brain metabolite alterations caused by pharmacological treatment. In fact, we found that these treatments reversed decreased brain levels of monoamine neurotransmitters, GABA, and anandamide caused by depression. For monoamine neurotransmitters, pharmacological treatment increased serotonin levels and decreased levels of its main metabolite 5-hydroxyindoleacetic acid, increased concentrations of dopamine and its metabolite 3-methoxytyramine, and upregulated norepinephrine levels. These findings are consistent with the neurochemical mechanism of classic antidepressants [34]. We also found that pharmacological medication promoted the synthesis of GABA and reduced the content of its precursor glutamic acid. However, human studies investigating alterations of glutamatergic metabolite levels after treatment reported inconsistent results. Previous magnetic resonance spectroscopy studies reported that both decreased glutamic acid levels and increased GABA levels in the cingulate were associated with reduced depressive symptoms in patients with major depressive disorder (MDD) following citalopram treatment [35, 36]. Another study found that cortical concentrations of these metabolites were not altered after escitalopram treatment [37]. We also found reduced glutamine levels in blood and urine following treatment, but discordant results were reported in patients with MDD [38, 39]. Therefore, further studies with larger sample sizes are needed to investigate alterations of glutamatergic metabolites induced by antidepressant treatment. We also found that pharmacological treatment increased anandamide levels in the brain. Consistent with our results, a preclinical study showed that anandamide exerted antidepressant effects by antagonizing the cannabinoid CB1 receptor [40].

Our results show that pharmacological treatment reduced corticosterone levels in blood and cortisol levels in urine. In rodents, cortisol and corticosterone are endogenous glucocorticoids induced by stressful conditions through activation of the hypothalamic-pituitary-adrenal (HPA) axis [41], disturbance of which have been implicated in the pathophysiology of depression [42]. In line with our results, preclinical and clinical evidence show that modulating the function of the HPA axis is a potential treatment approach for depression [43]. Therefore, the results of this study support the notion that pharmacological medications exert antidepressant effects by alleviating activation of the HPA axis.

In this study, we found that pharmacological treatment increased central and circulating levels of tryptophan, while brain levels of quinolinic acid, kynurenine, and the kynurenine/tryptophan ratio showed a decreasing trend or statistically significant decrease. Tryptophan and its two metabolites (indoleacetic acid and xanthurenic acid) were also upregulated in urine, suggesting that tryptophan levels were increased throughout the body after treatment. In line with this finding, recent meta-analyses suggested that disturbance of the tryptophan-kynurenine pathway is an important signature of patients with MDD [27, 44]. Quinolinic acid, a brain-endogenous metabolite synthesized from the tryptophan-kynurenine pathway by microglia, can activate the *N*-methyl-d-aspartate receptor to exert neurotoxic and oxidative actions [45]. Existing evidence suggests that immune activation promotes the transformation of tryptophan to kynurenine in the circulation, ultimately leading to the accumulation of quinolinic acid in the brain [46, 47]. Our current results show that pharmacological treatment reversed this process. Consistent with these findings, we found evidence that peripheral inflammation was alleviated following treatment. A large-scale meta-analysis identified *N*-acetyl glycoprotein as one of the inflammatory markers in patients with depression [48]. Herein, we found that pharmacological treatment reversed the elevation of *N*-acetyl glycoprotein levels in the blood of depression models. In addition, we found that pharmacological treatment reversed the elevation of myo-inositol in the brain, whereby it is regarded as a glial cell marker [49]. An in vivo study showed that the forced swimming test caused an elevation of myo-inositol in the prefrontal cortex of rats that could be counteracted by administration of the tricyclic antidepressant desipramine [50]. Taken together, our results suggest that pharmacological treatment may exert antidepressant effects by modulating disturbances of the tryptophan-kynurenine pathway and alleviating inflammatory activation.

Another important finding was that pharmacological treatment modulated microbiota-derived metabolites. We found that the levels of four short-chain fatty acids (SCFAs), including acetic acid, butyric acid, isovaleric acid, and propionic acid, were increased in feces and urine following treatment. SCFAs are mainly derived from dietary fiber and maintain the function of the innate gut barrier [51]. Preclinical evidence shows that supplementation with an SCFA mixture could counteract the depressive behavior caused by chronic stress [52]. Clinical evidence also demonstrated that fecal levels of SCFAs were negatively correlated with depression scale scores [53]. Therefore, pharmacological treatment may exert antidepressant effects by modulating SCFA-producing bacteria. We also found that blood and urine levels of betaine, which is derived from dietary choline, were increased following treatment [54]. Interestingly, a recent study showed that betaine supplementation in mice could lead to stress resilience accompanied by alleviated elevation of fecal SCFAs and release of circulating interleukin 6 [55]. Trimethylamine *N*-oxide is a gut-microbe-derived metabolite produced from dietary choline and carnitine [56]. In the current study, we found that pharmacological treatment alleviated the reduction of trimethylamine *N*-oxide in the blood of depression models, suggesting that these treatments could improve nutritional status and modulate gut microbiota. Phenylacetylglycine, a metabolite transformed from dietary phenylalanine that acts on adrenergic receptors, has been implicated in platelet hyperresponsiveness [57]. Interestingly, previous studies demonstrated that stress could lead to a prothrombotic state, which could be attenuated by antidepressants [58, 59]. We found that concentrations of phenylacetylglycine were decreased following treatment, suggesting that these treatments may also affect phenylacetylglycine synthesis to alleviate prothrombotic states by modulating gut microbiota.

The results of vote counting suggest that pharmacological treatment improved abnormalities of amino acid metabolism in animal models of depression. Branched-chain amino acids, including leucine, isoleucine, and valine, play important roles in promoting protein synthesis [60]. We found that pharmacological treatment elevated blood levels of these three branched-chain amino acids in this study, consistent with our previous finding that circulating levels of leucine were decreased in both patients with MDD and depression models, implicating a chronic catabolic state in depression [27, 31]. In addition, we found that ornithine was the only upregulated metabolite in the liver. Ornithine is synthesized as an intermediate metabolite in the urea cycle, which disposes excess nitrogen by converting ammonia to urea [61]. Collectively, this evidence suggests that pharmacological treatment could promote protein synthesis and improve disturbances of nitrogen metabolism.

We found that pharmacological medications modulated energy metabolism in animal models of depression. The results of vote counting showed that pharmacological treatment reduced blood glucose levels and alleviated the accumulation of lactic acid in the brain and periphery. One possible reason for this observation is that stress conditions in animal models promoted insulin resistance and mobilized glucose [62], which were reversed by pharmacological treatment. Moreover, circulating levels of two energy metabolites, oxoglutaric acid, and creatine, were elevated after treatment. Oxoglutaric acid, also known as alpha-ketoglutarate, is an intermediate in the citric acid cycle [63]. Creatine is synthesized in the liver and transported throughout the body to facilitate the recycling of adenosine triphosphate, and creatine supplementation exerted antidepressant effects in mice [64, 65]. Therefore, these results implicate improvements in energy dyshomeostasis in the molecular mechanism of pharmacological treatments of depression.

Our findings also suggest that pharmacological medication improved peripheral lipid abnormalities in depression models. Following treatment, LDL levels were elevated and HDL levels were reduced in blood. A previous study demonstrated that antidepressant therapy decreased blood LDL levels without affecting HDL levels [66], while another study found that patients with MDD presenting with and without metabolic syndrome showed different trends for these metabolic parameters after antidepressant treatment [67]. A previous study with 2461 participants also showed that patients with different MDD subtypes had distinct blood lipid profiles [68]. Therefore, more studies are still needed to explore baseline levels and post-treatment changes in LDL and HDL cholesterols in the context of depression. We also found that levels of arachidonic acid, an essential omega-6 fatty acid, were decreased in the blood and liver. Clinical studies previously found that the reduction of arachidonic acid in blood was associated with antidepressant treatment response [69]. Moreover, because arachidonic acid is a precursor of pro-inflammatory eicosanoids, its reduction suggests abrogation of the inflammatory response in depression [70].

Finally, the present study compared metabolomic changes associated with different pharmacological treatments in depression models. The results of vote-counting procedures showed that both antidepressants and non-antidepressants modulated levels of monoamine neurotransmitters and GABA in the brain. However, the regulatory effect of antidepressants on anandamide was unclear. Moreover, we found that antidepressants increased central and peripheral levels of tryptophan, while non-antidepressants did not show similar effects. Notably, we found that most of the dysregulated metabolites in the blood resulting from antidepressants were also altered by non-antidepressants, suggesting a significant overlap in the metabolic changes elicited by antidepressants and non-antidepressants, although the latter caused more diverse molecular changes. Despite these findings, more studies are needed to further verify our results.

## Limitations

There are some limitations to this study. First, we could not identify dysregulated metabolites other than the candidate metabolites due to the shortcomings of the vote-counting method. Second, although more comprehensive statistical results could be provided by merging raw data or combining mean concentrations of the candidate metabolites, this remains a difficult task to complete. In addition, it is difficult to compare metabolomic changes brought about by different types of pharmacological treatments, such as fluoxetine and venlafaxine, due to the limited number of metabolite entries.

## Conclusions

We present a systematic characterization of metabolomic alterations resulting from pharmacological treatment in depression models, which contributes to the understanding of the molecular mechanism of antidepressant effects. The results of vote-counting procedures suggested that pharmacological medications reversed the reduction of brain neurotransmitter levels caused by depression, modulated disturbance of the tryptophan-kynurenine pathway and inflammatory activation, and alleviated abnormalities of amino acid, energy, and lipid metabolism. Pharmacological treatment reversed metabolite disturbances induced by depression, however further studies with more metabolite entries are needed to verify our results.

## Supplementary information

Supplemental Material

Dataset 1

Dataset 2

Dataset 3

Dataset 4
