# Supplementary material for: Effects of pharmacological treatment on metabolomic alterations in animal models of depression
Source: Transl Psychiatry. 2022 Apr 29;12:175. doi: 10.1038/s41398-022-01947-5 (PMC9055046; doi:10.1038/s41398-022-01947-5)
Supplement: Supplementary file 1 — Supplemental Material [file 41398_2022_1947_MOESM1_ESM.docx]

**Effects of pharmacological treatment on metabolomic alterations in animal models of depression**

This article contains Supplementary Tables 1–20 and Supplementary Data 1–4.

This article does not contain Supplementary Figures.

**List of Supplementary Tables**

Supplementary Table 1. Numbers of studies and differential metabolites excluded from MENDA database.

Supplementary Table 2. Summary of the numbers of studies and metabolite entries.

Supplementary Table 3. Vote-counting results for the brain.

Supplementary Table 4. Vote-counting results for the hippocampus.

Supplementary Table 5. Vote-counting results for the prefrontal cortex.

Supplementary Table 6. Vote-counting results for the hypothalamus.

Supplementary Table 7. Vote-counting results for blood.

Supplementary Table 8. Vote-counting results for plasma.

Supplementary Table 9. Vote-counting results for serum.

Supplementary Table 10. Vote-counting results for urine.

Supplementary Table 11. Vote-counting results for the liver.

Supplementary Table 12. Vote-counting results for feces.

Supplementary Table 13. Vote-counting results for the brain in antidepressant.

Supplementary Table 14. Vote-counting results for the brain in non-antidepressant.

Supplementary Table 15. Vote-counting results for blood in antidepressant.

Supplementary Table 16. Vote-counting results for blood in non-antidepressant.

Supplementary Table 17. Vote-counting results for urine in antidepressant.

Supplementary Table 18. Vote-counting results for urine in non-antidepressant.

Supplementary Table 19. Vote-counting results for the liver in non-antidepressant.

Supplementary Table 20. Vote-counting results for feces in non-antidepressant.

**List of Supplementary Data**

Supplementary Data 1. Full-text excluded articles.

Supplementary Data 2. Studies excluded from MENDA database.

Supplementary Data 3. The full information of included studies.

Supplementary Data 4. The full list of candidate metabolites.

| **Supplementary Table 1. Numbers of studies and differential metabolites excluded from MENDA database.** | | |
| --- | --- | --- |
| **Category** | **No. of excluded studies** | **No. of excluded metabolites** |
| Other type of study (depression vs. control) | 457 | 5,477 |
| Other type of study (healthy individuals) | 65 | 1,316 |
| Other type of study (responder vs. nonresponder) | 6 | 15 |
| Human study | 104 | 2,669 |
| Non-human primate model study | 4 | 714 |
| Magnetic resonance spectroscopy study | 8 | 35 |
| Non-pharmacological treatment | 8 | 93 |
| Other type of tissue | 8 | 36 |
| **Total** | **660** | **10,355** |

| **Supplementary Table 2. Summary of the numbers of studies and metabolite entries.** | | | | |
| --- | --- | --- | --- | --- |
| **Tissue** | **No. of studies (n = 157)** | **No. of metabolite entries (n = 2,757)** | | |
|  |  | **All** | **Antidepressant** | **Non-antidepressant** |
| Brain | 76 | 899 | 344 | 555 |
| Blood | 62 | 1,007 | 289 | 718 |
| Urine | 23 | 518 | 85 | 433 |
| Liver | 9 | 210 | 59 | 151 |
| Feces | 16 | 123 | 26 | 97 |

| **Supplementary Table 3. Vote-counting results for the brain.** | | | | | |
| --- | --- | --- | --- | --- | --- |
| **Metabolites** | **Vote-counting statistic** | **No. of studies that report on the metabolite** | | | ***P* value** |
|  |  | **All** | **Upregulated** | **Downregulated** |  |
| Serotonin | 47 | 59 | 53 | 6 | <0.001 |
| Dopamine | 41 | 41 | 41 | 0 | <0.001 |
| Norepinephrine | 32 | 38 | 35 | 3 | <0.001 |
| Gamma-Aminobutyric acid | 31 | 47 | 39 | 8 | <0.001 |
| Anandamide | 11 | 11 | 11 | 0 | <0.001 |
| Hypoxanthine | 9 | 9 | 9 | 0 | 0.002 |
| L-Tryptophan | 9 | 15 | 12 | 3 | 0.018 |
| N-Acetyl-L-aspartic acid | 6 | 14 | 10 | 4 | 0.090 |
| 3-Methoxytyramine | 5 | 5 | 5 | 0 | 0.031 |
| Glycine | 5 | 9 | 7 | 2 | 0.090 |
| Epinephrine | 4 | 4 | 4 | 0 | 0.063 |
| Methoxyepinephrine | 4 | 4 | 4 | 0 | 0.063 |
| Oleoylethanolamide | 4 | 4 | 4 | 0 | 0.063 |
| Docosahexaenoic acid | 3 | 5 | 4 | 1 | 0.188 |
| Homovanillic acid | 3 | 5 | 4 | 1 | 0.188 |
| L-Dopa | 3 | 5 | 4 | 1 | 0.188 |
| L-Isoleucine | 3 | 5 | 4 | 1 | 0.188 |
| L-Valine | 3 | 7 | 5 | 2 | 0.227 |
| 3,4-Dihydroxybenzeneacetic acid | 2 | 4 | 3 | 1 | 0.313 |
| L-Glutamine | 2 | 14 | 8 | 6 | 0.395 |
| L-Leucine | 2 | 6 | 4 | 2 | 0.344 |
| L-Phenylalanine | 2 | 14 | 8 | 6 | 0.395 |
| Oleic acid | 2 | 4 | 3 | 1 | 0.313 |
| Taurine | 2 | 8 | 5 | 3 | 0.363 |
| Acetylcholine | 1 | 5 | 3 | 2 | 0.500 |
| Indoleacrylic acid | 1 | 5 | 3 | 2 | 0.500 |
| L-Tyrosine | 1 | 11 | 6 | 5 | 0.500 |
| L-Methionine | 0 | 4 | 2 | 2 | 0.688 |
| 5-Hydroxytryptophol | −1 | 7 | 3 | 4 | 0.500 |
| Arachidonic acid | −1 | 7 | 3 | 4 | 0.500 |
| Creatine | −1 | 5 | 2 | 3 | 0.500 |
| L-Aspartic acid | −1 | 5 | 2 | 3 | 0.500 |
| Succinic acid | −1 | 5 | 2 | 3 | 0.500 |
| 3-Hydroxyanthranilic acid | −2 | 4 | 1 | 3 | 0.313 |
| 5-Hydroxy-L-tryptophan | −2 | 6 | 2 | 4 | 0.344 |
| Creatinine | −2 | 4 | 1 | 3 | 0.313 |
| L-Alanine | −2 | 4 | 1 | 3 | 0.313 |
| LysoPC(16:0) | −2 | 6 | 2 | 4 | 0.344 |
| Glutathione | −4 | 6 | 1 | 5 | 0.109 |
| Inosine | −4 | 12 | 4 | 8 | 0.194 |
| Kynurenine/tryptophan ratio | −5 | 5 | 0 | 5 | 0.031 |
| L-Lactic acid | −5 | 5 | 0 | 5 | 0.031 |
| L-Kynurenine | −6 | 10 | 2 | 8 | 0.055 |
| myo-Inositol | −8 | 8 | 0 | 8 | 0.004 |
| 5-Hydroxyindoleacetic acid | −13 | 31 | 9 | 22 | 0.015 |
| L-Glutamic acid | −14 | 48 | 17 | 31 | 0.030 |
| Quinolinic acid | −18 | 18 | 0 | 18 | <0.001 |
| *LysoPC*, lysophosphatidylcholine. | | | | | |

| **Supplementary Table 4. Vote-counting results for the hippocampus.** | | | | | |
| --- | --- | --- | --- | --- | --- |
| **Metabolites** | **Vote-counting statistic** | **No. of studies that report on the metabolite** | | | ***P* value** |
|  |  | **All** | **Upregulated** | **Downregulated** |  |
| Serotonin | 23 | 23 | 23 | 0 | <0.001 |
| Gamma-Aminobutyric acid | 17 | 17 | 17 | 0 | <0.001 |
| Norepinephrine | 13 | 13 | 13 | 0 | <0.001 |
| Dopamine | 12 | 12 | 12 | 0 | <0.001 |
| Glycine | 7 | 7 | 7 | 0 | 0.008 |
| Hypoxanthine | 7 | 7 | 7 | 0 | 0.008 |
| L-Tryptophan | 6 | 12 | 9 | 3 | 0.073 |
| N-Acetyl-L-aspartic acid | 6 | 10 | 8 | 2 | 0.055 |
| L-Glutamine | 3 | 7 | 5 | 2 | 0.227 |
| Homovanillic acid | 2 | 4 | 3 | 1 | 0.313 |
| L-Leucine | 2 | 6 | 4 | 2 | 0.344 |
| L-Valine | 2 | 4 | 3 | 1 | 0.313 |
| Oleic acid | 2 | 4 | 3 | 1 | 0.313 |
| Indoleacrylic acid | 1 | 5 | 3 | 2 | 0.500 |
| L-Phenylalanine | 1 | 11 | 6 | 5 | 0.500 |
| Taurine | 1 | 5 | 3 | 2 | 0.500 |
| LysoPC(16:0) | −1 | 5 | 2 | 3 | 0.500 |
| Creatinine | −2 | 4 | 1 | 3 | 0.313 |
| L-Tyrosine | −2 | 8 | 3 | 5 | 0.363 |
| Succinic acid | −2 | 4 | 1 | 3 | 0.313 |
| Glutathione | −4 | 6 | 1 | 5 | 0.109 |
| L-Kynurenine | −4 | 8 | 2 | 6 | 0.145 |
| Quinolinic acid | −4 | 4 | 0 | 4 | 0.063 |
| 5-Hydroxyindoleacetic acid | −5 | 11 | 3 | 8 | 0.113 |
| L-Lactic acid | −5 | 5 | 0 | 5 | 0.031 |
| Inosine | −6 | 10 | 2 | 8 | 0.055 |
| myo-Inositol | −8 | 8 | 0 | 8 | 0.004 |
| L-Glutamic acid | −9 | 23 | 7 | 16 | 0.047 |
| *LysoPC*, lysophosphatidylcholine. | | | | | |

| **Supplementary Table 5. Vote-counting results for the prefrontal cortex.** | | | | | |
| --- | --- | --- | --- | --- | --- |
| **Metabolites** | **Vote-counting statistic** | **No. of studies that report on the metabolite** | | | ***P* value** |
|  |  | **All** | **Upregulated** | **Downregulated** |  |
| Serotonin | 12 | 12 | 12 | 0 | <0.001 |
| Norepinephrine | 10 | 10 | 10 | 0 | 0.001 |
| Gamma-Aminobutyric acid | 7 | 9 | 8 | 1 | 0.020 |
| Dopamine | 6 | 6 | 6 | 0 | 0.016 |
| L-Glutamine | 2 | 4 | 3 | 1 | 0.313 |
| L-Isoleucine | 2 | 4 | 3 | 1 | 0.313 |
| 5-Hydroxytryptophol | −3 | 5 | 1 | 4 | 0.188 |
| L-Glutamic acid | −3 | 9 | 3 | 6 | 0.254 |

| **Supplementary Table 6. Vote-counting results for the hypothalamus.** | | | | | |
| --- | --- | --- | --- | --- | --- |
| **Metabolites** | **Vote-counting statistic** | **No. of studies that report on the metabolite** | | | ***P* value** |
|  |  | **All** | **Upregulated** | **Downregulated** |  |
| Dopamine | 5 | 5 | 5 | 0 | 0.031 |
| Gamma-Aminobutyric acid | 5 | 5 | 5 | 0 | 0.031 |
| Serotonin | 5 | 5 | 5 | 0 | 0.031 |
| Norepinephrine | 4 | 4 | 4 | 0 | 0.063 |
| 5-Hydroxyindoleacetic acid | 1 | 5 | 3 | 2 | 0.500 |
| L-Glutamic acid | −3 | 5 | 1 | 4 | 0.188 |

| **Supplementary Table 7. Vote-counting results for blood.** | | | | | |
| --- | --- | --- | --- | --- | --- |
| **Metabolites** | **Vote-counting statistic** | **No. of studies that report on the metabolite** | | | ***P* value** |
|  |  | **All** | **Upregulated** | **Downregulated** |  |
| Trimethylamine N-oxide | 29 | 33 | 31 | 2 | <0.001 |
| L-Isoleucine | 21 | 23 | 22 | 1 | <0.001 |
| L-Leucine | 16 | 16 | 16 | 0 | <0.001 |
| L-Tryptophan | 12 | 18 | 15 | 3 | 0.004 |
| Creatine | 11 | 25 | 18 | 7 | 0.022 |
| Serotonin | 10 | 14 | 12 | 2 | 0.006 |
| L-Valine | 9 | 11 | 10 | 1 | 0.006 |
| Betaine | 8 | 10 | 9 | 1 | 0.011 |
| Pyruvic acid | 7 | 15 | 11 | 4 | 0.059 |
| LDL | 6 | 6 | 6 | 0 | 0.016 |
| Indoleacrylic acid | 5 | 7 | 6 | 1 | 0.063 |
| Indoxyl sulfate | 4 | 4 | 4 | 0 | 0.063 |
| L-Acetylcarnitine | 4 | 4 | 4 | 0 | 0.063 |
| L-Phenylalanine | 4 | 14 | 9 | 5 | 0.212 |
| L-Proline | 4 | 6 | 5 | 1 | 0.109 |
| LysoPC(18:0) | 4 | 4 | 4 | 0 | 0.063 |
| unsaturated lipids L17 | 4 | 4 | 4 | 0 | 0.063 |
| unsaturated lipids L19 | 4 | 4 | 4 | 0 | 0.063 |
| L-Tyrosine | 3 | 5 | 4 | 1 | 0.188 |
| Palmitic acid | 3 | 5 | 4 | 1 | 0.188 |
| Citric acid | 2 | 10 | 6 | 4 | 0.377 |
| Citronellyl anthranilate | 2 | 4 | 3 | 1 | 0.313 |
| Gamma-Aminobutyric acid | 2 | 4 | 3 | 1 | 0.313 |
| Hippuric acid | 2 | 6 | 4 | 2 | 0.344 |
| L-Palmitoylcarnitine | 2 | 4 | 3 | 1 | 0.313 |
| Cholic acid | 1 | 9 | 5 | 4 | 0.500 |
| L-Threonine | 1 | 7 | 4 | 3 | 0.500 |
| Sphingosine 1-phosphate | 1 | 5 | 3 | 2 | 0.500 |
| L-Glutamic acid | 0 | 16 | 8 | 8 | 0.598 |
| L-Methionine | 0 | 8 | 4 | 4 | 0.637 |
| LysoPC(18:2(9Z,12Z)) | 0 | 4 | 2 | 2 | 0.688 |
| Succinic acid | 0 | 4 | 2 | 2 | 0.688 |
| LysoPC(16:0) | −1 | 11 | 5 | 6 | 0.500 |
| LysoPC(20:4(5Z,8Z,11Z,14Z)) | −1 | 5 | 2 | 3 | 0.500 |
| LysoPE(0:0/20:2(11Z,14Z)) | −1 | 5 | 2 | 3 | 0.500 |
| Sphingosine | −1 | 5 | 2 | 3 | 0.500 |
| Deoxycholic acid | −2 | 6 | 2 | 4 | 0.344 |
| D-Glucose | −2 | 8 | 3 | 5 | 0.363 |
| Phytosphingosine | −2 | 6 | 2 | 4 | 0.344 |
| Lipid | −3 | 9 | 3 | 6 | 0.254 |
| Quinolinic acid | −3 | 5 | 1 | 4 | 0.188 |
| Sphinganine | −3 | 9 | 3 | 6 | 0.254 |
| D-Fructose | −4 | 4 | 0 | 4 | 0.063 |
| Glycerol | −4 | 4 | 0 | 4 | 0.063 |
| L-Lysine | −4 | 4 | 0 | 4 | 0.063 |
| LysoPC(15:0) | −4 | 6 | 1 | 5 | 0.109 |
| Oxoglutaric acid | −4 | 4 | 0 | 4 | 0.063 |
| Allantoin | −5 | 5 | 0 | 5 | 0.031 |
| Taurine | −5 | 5 | 0 | 5 | 0.031 |
| Choline | −6 | 26 | 10 | 16 | 0.164 |
| 3-Hydroxybutyric acid | −7 | 15 | 4 | 11 | 0.059 |
| Arachidonic acid | −7 | 13 | 3 | 10 | 0.046 |
| myo-Inositol | −7 | 7 | 0 | 7 | 0.008 |
| Glycine | −8 | 10 | 1 | 9 | 0.011 |
| HDL | −8 | 8 | 0 | 8 | 0.004 |
| L-Alanine | −9 | 15 | 3 | 12 | 0.018 |
| Phenylacetylglycine | −9 | 9 | 0 | 9 | 0.002 |
| Beta-D-Glucose | −11 | 11 | 0 | 11 | <0.001 |
| Corticosterone | −11 | 11 | 0 | 11 | <0.001 |
| L-Glutamine | −15 | 15 | 0 | 15 | <0.001 |
| N-acetyl glycoprotein | −18 | 26 | 4 | 22 | <0.001 |
| L-Lactic acid | −23 | 27 | 2 | 25 | <0.001 |
| Alpha-D-Glucose | −25 | 31 | 3 | 28 | <0.001 |
| *LDL*, low-density lipoprotein; *LysoPC*, lysophosphatidylcholine; *LysoPE*, lysophosphatidylethanolamine; *HDL*, high-density lipoprotein. | | | | | |

| **Supplementary Table 8. Vote-counting results for plasma.** | | | | | |
| --- | --- | --- | --- | --- | --- |
| **Metabolites** | **Vote-counting statistic** | **No. of studies that report on the metabolite** | | | ***P* value** |
|  |  | **All** | **Upregulated** | **Downregulated** |  |
| L-Tryptophan | 10 | 10 | 10 | 0 | 0.001 |
| N-acetyl glycoprotein | 4 | 4 | 4 | 0 | 0.063 |
| Palmitic acid | 4 | 4 | 4 | 0 | 0.063 |
| unsaturated lipids L17 | 4 | 4 | 4 | 0 | 0.063 |
| unsaturated lipids L19 | 4 | 4 | 4 | 0 | 0.063 |
| Serotonin | 3 | 7 | 5 | 2 | 0.227 |
| Gamma-Aminobutyric acid | 2 | 4 | 3 | 1 | 0.313 |
| L-Valine | 2 | 4 | 3 | 1 | 0.313 |
| L-Phenylalanine | 1 | 7 | 4 | 3 | 0.500 |
| Citric acid | −2 | 4 | 1 | 3 | 0.313 |
| Creatine | −2 | 6 | 2 | 4 | 0.344 |
| L-Glutamic acid | −2 | 8 | 3 | 5 | 0.363 |
| LysoPC(16:0) | −2 | 4 | 1 | 3 | 0.313 |
| 3-Hydroxybutyric acid | −3 | 5 | 1 | 4 | 0.188 |
| Pyruvic acid | −3 | 5 | 1 | 4 | 0.188 |
| Quinolinic acid | −3 | 5 | 1 | 4 | 0.188 |
| Alpha-D-Glucose | −4 | 4 | 0 | 4 | 0.063 |
| Beta-D-Glucose | −4 | 4 | 0 | 4 | 0.063 |
| D-Fructose | −4 | 4 | 0 | 4 | 0.063 |
| L-Lysine | −4 | 4 | 0 | 4 | 0.063 |
| L-Methionine | −4 | 4 | 0 | 4 | 0.063 |
| myo-Inositol | −4 | 4 | 0 | 4 | 0.063 |
| Phenylacetylglycine | −4 | 4 | 0 | 4 | 0.063 |
| Sphinganine | −4 | 4 | 0 | 4 | 0.063 |
| D-Glucose | −5 | 5 | 0 | 5 | 0.031 |
| L-Glutamine | −5 | 5 | 0 | 5 | 0.031 |
| Glycine | −6 | 8 | 1 | 7 | 0.035 |
| Arachidonic acid | −7 | 7 | 0 | 7 | 0.008 |
| Corticosterone | −11 | 11 | 0 | 11 | <0.001 |
| *LysoPC*, lysophosphatidylcholine. | | | | | |

| **Supplementary Table 9. Vote-counting results for serum.** | | | | | |
| --- | --- | --- | --- | --- | --- |
| **Metabolites** | **Vote-counting statistic** | **No. of studies that report on the metabolite** | | | ***P* value** |
|  |  | **All** | **Upregulated** | **Downregulated** |  |
| Trimethylamine N-oxide | 28 | 32 | 30 | 2 | <0.001 |
| L-Isoleucine | 21 | 23 | 22 | 1 | <0.001 |
| Creatine | 13 | 19 | 16 | 3 | 0.002 |
| L-Leucine | 13 | 13 | 13 | 0 | <0.001 |
| Pyruvic acid | 10 | 10 | 10 | 0 | 0.001 |
| Betaine | 7 | 9 | 8 | 1 | 0.020 |
| L-Valine | 7 | 7 | 7 | 0 | 0.008 |
| Serotonin | 7 | 7 | 7 | 0 | 0.008 |
| Indoleacrylic acid | 5 | 7 | 6 | 1 | 0.063 |
| Citric acid | 4 | 6 | 5 | 1 | 0.109 |
| Indoxyl sulfate | 4 | 4 | 4 | 0 | 0.063 |
| L-Methionine | 4 | 4 | 4 | 0 | 0.063 |
| LysoPC(18:0) | 4 | 4 | 4 | 0 | 0.063 |
| L-Phenylalanine | 3 | 7 | 5 | 2 | 0.227 |
| L-Proline | 3 | 5 | 4 | 1 | 0.188 |
| L-Glutamic acid | 2 | 8 | 5 | 3 | 0.363 |
| L-Tryptophan | 2 | 8 | 5 | 3 | 0.363 |
| LysoPC(16:0) | 1 | 7 | 4 | 3 | 0.500 |
| Sphinganine | 1 | 5 | 3 | 2 | 0.500 |
| Sphingosine 1-phosphate | 1 | 5 | 3 | 2 | 0.500 |
| Arachidonic acid | 0 | 6 | 3 | 3 | 0.656 |
| Hippuric acid | 0 | 4 | 2 | 2 | 0.688 |
| LysoPC(18:2(9Z,12Z)) | 0 | 4 | 2 | 2 | 0.688 |
| Phytosphingosine | 0 | 4 | 2 | 2 | 0.688 |
| Sphingosine | −1 | 5 | 2 | 3 | 0.500 |
| Cholic acid | −2 | 6 | 2 | 4 | 0.344 |
| L-Threonine | −2 | 4 | 1 | 3 | 0.313 |
| Lipid | −3 | 9 | 3 | 6 | 0.254 |
| 3-Hydroxybutyric acid | −4 | 10 | 3 | 7 | 0.172 |
| Deoxycholic acid | −4 | 4 | 0 | 4 | 0.063 |
| LysoPC(15:0) | −4 | 6 | 1 | 5 | 0.109 |
| Oxoglutaric acid | −4 | 4 | 0 | 4 | 0.063 |
| Allantoin | −5 | 5 | 0 | 5 | 0.031 |
| Choline | −5 | 25 | 10 | 15 | 0.212 |
| Phenylacetylglycine | −5 | 5 | 0 | 5 | 0.031 |
| Taurine | −5 | 5 | 0 | 5 | 0.031 |
| Beta-D-Glucose | −7 | 7 | 0 | 7 | 0.008 |
| HDL | −8 | 8 | 0 | 8 | 0.004 |
| L-Alanine | −8 | 14 | 3 | 11 | 0.029 |
| L-Glutamine | −10 | 10 | 0 | 10 | 0.001 |
| Alpha-D-Glucose | −21 | 27 | 3 | 24 | <0.001 |
| L-Lactic acid | −21 | 25 | 2 | 23 | <0.001 |
| N-acetyl glycoprotein | −22 | 22 | 0 | 22 | <0.001 |
| *LysoPC*, lysophosphatidylcholine; *HDL*, high-density lipoprotein. | | | | | |

| **Supplementary Table 10. Vote-counting results for urine.** | | | | | |
| --- | --- | --- | --- | --- | --- |
| **Metabolites** | **Vote-counting statistic** | **No. of studies that report on the metabolite** | | | ***P* value** |
|  |  | **All** | **Upregulated** | **Downregulated** |  |
| Oxoglutaric acid | 12 | 12 | 12 | 0 | <0.001 |
| Acetic acid | 10 | 10 | 10 | 0 | 0.001 |
| Creatinine | 9 | 13 | 11 | 2 | 0.011 |
| Betaine | 8 | 8 | 8 | 0 | 0.004 |
| Citric acid | 6 | 22 | 14 | 8 | 0.143 |
| Phenylacetylglycine | 6 | 6 | 6 | 0 | 0.016 |
| Xanthurenic acid | 6 | 8 | 7 | 1 | 0.035 |
| Creatine | 5 | 5 | 5 | 0 | 0.031 |
| Indoleacetic acid | 5 | 5 | 5 | 0 | 0.031 |
| Kynurenic acid | 5 | 11 | 8 | 3 | 0.113 |
| L-Tryptophan | 5 | 5 | 5 | 0 | 0.031 |
| Oxalic acid | 4 | 4 | 4 | 0 | 0.063 |
| Palmitic acid | 4 | 4 | 4 | 0 | 0.063 |
| Pimelic acid | 4 | 4 | 4 | 0 | 0.063 |
| Uric acid | 4 | 4 | 4 | 0 | 0.063 |
| Succinic acid | 3 | 9 | 6 | 3 | 0.254 |
| Allantoin | 2 | 4 | 3 | 1 | 0.313 |
| L-Asparagine | 2 | 4 | 3 | 1 | 0.313 |
| L-Proline | 2 | 6 | 4 | 2 | 0.344 |
| 3-Hydroxyhippuric acid | 0 | 4 | 2 | 2 | 0.688 |
| L-Serine | 0 | 4 | 2 | 2 | 0.688 |
| Trimethylamine | 0 | 8 | 4 | 4 | 0.637 |
| L-Phenylalanine | −1 | 9 | 4 | 5 | 0.500 |
| Cyclic AMP | −2 | 4 | 1 | 3 | 0.313 |
| L-Tyrosine | −2 | 8 | 3 | 5 | 0.363 |
| Pyruvic acid | −2 | 8 | 3 | 5 | 0.363 |
| Taurine | −2 | 8 | 3 | 5 | 0.363 |
| Hippuric acid | −3 | 17 | 7 | 10 | 0.315 |
| L-Glutamic acid | −3 | 5 | 1 | 4 | 0.188 |
| Dimethylamine | −4 | 4 | 0 | 4 | 0.063 |
| L-Glutamine | −5 | 5 | 0 | 5 | 0.031 |
| Cortisol | −6 | 6 | 0 | 6 | 0.016 |
| Glycine | −7 | 7 | 0 | 7 | 0.008 |
| *AMP*, adenosine monophosphate. | | | | | |

| **Supplementary Table 11. Vote-counting results for the liver.** | | | | | |
| --- | --- | --- | --- | --- | --- |
| **Metabolites** | **Vote-counting statistic** | **No. of studies that report on the metabolite** | | | ***P* value** |
|  |  | **All** | **Upregulated** | **Downregulated** |  |
| Ornithine | 6 | 6 | 6 | 0 | 0.016 |
| L-Glutamic acid | 5 | 13 | 9 | 4 | 0.133 |
| Glutathione | 4 | 8 | 6 | 2 | 0.145 |
| L-Lysine | 4 | 8 | 6 | 2 | 0.145 |
| Oxoglutaric acid | 2 | 8 | 5 | 3 | 0.363 |
| Glycerol | 1 | 5 | 3 | 2 | 0.500 |
| Succinic acid | −2 | 4 | 1 | 3 | 0.313 |
| Taurochenodesoxycholic acid | −2 | 6 | 2 | 4 | 0.344 |
| Ascorbic acid | −4 | 4 | 0 | 4 | 0.063 |
| Citric acid | −4 | 4 | 0 | 4 | 0.063 |
| 3'-AMP | −5 | 5 | 0 | 5 | 0.031 |
| Creatine | −5 | 5 | 0 | 5 | 0.031 |
| L-Tyrosine | −5 | 5 | 0 | 5 | 0.031 |
| Alpha-D-Glucose | −6 | 6 | 0 | 6 | 0.016 |
| L-Tryptophan | −6 | 6 | 0 | 6 | 0.016 |
| Arachidonic acid | −7 | 7 | 0 | 7 | 0.008 |
| Beta-D-Glucose | −8 | 8 | 0 | 8 | 0.004 |
| L-Lactic acid | −8 | 10 | 1 | 9 | 0.011 |
| *AMP*, adenosine monophosphate. | | | | | |

| **Supplementary Table 12. Vote-counting results for feces.** | | | | | |
| --- | --- | --- | --- | --- | --- |
| **Metabolites** | **Vote-counting statistic** | **No. of studies that report on the metabolite** | | | ***P* value** |
|  |  | **All** | **Upregulated** | **Downregulated** |  |
| Butyric acid | 8 | 10 | 9 | 1 | 0.011 |
| Acetic acid | 7 | 7 | 7 | 0 | 0.008 |
| Propionic acid | 7 | 11 | 9 | 2 | 0.033 |
| Isovaleric acid | 5 | 5 | 5 | 0 | 0.031 |
| Valeric acid | 4 | 4 | 4 | 0 | 0.063 |

| **Supplementary Table 13. Vote-counting results for the brain in antidepressant.** | | | | | |
| --- | --- | --- | --- | --- | --- |
| **Metabolites** | **Vote-counting statistic** | **No. of studies that report on the metabolite** | | | ***P* value** |
|  |  | **All** | **Upregulated** | **Downregulated** |  |
| Norepinephrine | 11 | 11 | 11 | 0 | <0.001 |
| Dopamine | 10 | 10 | 10 | 0 | 0.001 |
| Gamma-Aminobutyric acid | 8 | 12 | 10 | 2 | 0.019 |
| Serotonin | 8 | 20 | 14 | 6 | 0.058 |
| L-Tryptophan | 6 | 8 | 7 | 1 | 0.035 |
| L-Tyrosine | 3 | 7 | 5 | 2 | 0.227 |
| N-Acetyl-L-aspartic acid | 1 | 7 | 4 | 3 | 0.500 |
| L-Glutamine | 0 | 6 | 3 | 3 | 0.656 |
| L-Phenylalanine | 0 | 6 | 3 | 3 | 0.656 |
| Inosine | −1 | 5 | 2 | 3 | 0.500 |
| L-Kynurenine | −2 | 4 | 1 | 3 | 0.313 |
| 5-Hydroxy-L-tryptophan | −3 | 5 | 1 | 4 | 0.188 |
| L-Glutamic acid | −5 | 11 | 3 | 8 | 0.113 |
| 5-Hydroxyindoleacetic acid | −13 | 21 | 4 | 17 | 0.004 |
| Quinolinic acid | −17 | 17 | 0 | 17 | <0.001 |

| **Supplementary Table 14. Vote-counting results for the brain in non-antidepressant.** | | | | | |
| --- | --- | --- | --- | --- | --- |
| **Metabolites** | **Vote-counting statistic** | **No. of studies that report on the metabolite** | | | ***P* value** |
|  |  | **All** | **Upregulated** | **Downregulated** |  |
| Serotonin | 39 | 39 | 39 | 0 | <0.001 |
| Dopamine | 31 | 31 | 31 | 0 | <0.001 |
| Gamma-Aminobutyric acid | 23 | 35 | 29 | 6 | <0.001 |
| Norepinephrine | 21 | 27 | 24 | 3 | <0.001 |
| Anandamide | 11 | 11 | 11 | 0 | <0.001 |
| Hypoxanthine | 6 | 6 | 6 | 0 | 0.016 |
| N-Acetyl-L-aspartic acid | 5 | 7 | 6 | 1 | 0.063 |
| L-Tryptophan | 3 | 7 | 5 | 2 | 0.227 |
| 3,4-Dihydroxybenzeneacetic acid | 2 | 4 | 3 | 1 | 0.313 |
| Glycine | 2 | 6 | 4 | 2 | 0.344 |
| L-Glutamine | 2 | 8 | 5 | 3 | 0.363 |
| L-Phenylalanine | 2 | 8 | 5 | 3 | 0.363 |
| Taurine | 1 | 7 | 4 | 3 | 0.500 |
| 5-Hydroxyindoleacetic acid | 0 | 10 | 5 | 5 | 0.623 |
| 5-Hydroxytryptophol | 0 | 6 | 3 | 3 | 0.656 |
| L-Methionine | 0 | 4 | 2 | 2 | 0.688 |
| L-Valine | 0 | 4 | 2 | 2 | 0.688 |
| Arachidonic acid | −1 | 5 | 2 | 3 | 0.500 |
| 3-Hydroxyanthranilic acid | −2 | 4 | 1 | 3 | 0.313 |
| L-Tyrosine | −2 | 4 | 1 | 3 | 0.313 |
| Succinic acid | −2 | 4 | 1 | 3 | 0.313 |
| Inosine | −3 | 7 | 2 | 5 | 0.227 |
| L-Kynurenine | −4 | 6 | 1 | 5 | 0.109 |
| L-Lactic acid | −4 | 4 | 0 | 4 | 0.063 |
| myo-Inositol | −5 | 5 | 0 | 5 | 0.031 |
| L-Glutamic acid | −9 | 37 | 14 | 23 | 0.094 |

| **Supplementary Table 15. Vote-counting results for blood in antidepressant.** | | | | | |
| --- | --- | --- | --- | --- | --- |
| **Metabolites** | **Vote-counting statistic** | **No. of studies that report on the metabolite** | | | ***P* value** |
|  |  | **All** | **Upregulated** | **Downregulated** |  |
| Trimethylamine N-oxide | 7 | 9 | 8 | 1 | 0.020 |
| L-Leucine | 6 | 6 | 6 | 0 | 0.016 |
| L-Tryptophan | 6 | 8 | 7 | 1 | 0.035 |
| Creatine | 2 | 6 | 4 | 2 | 0.344 |
| Serotonin | 2 | 6 | 4 | 2 | 0.344 |
| L-Glutamic acid | −1 | 5 | 2 | 3 | 0.500 |
| LysoPC(16:0) | −1 | 5 | 2 | 3 | 0.500 |
| Choline | −2 | 8 | 3 | 5 | 0.363 |
| Glycine | −2 | 4 | 1 | 3 | 0.313 |
| Lipid | −2 | 4 | 1 | 3 | 0.313 |
| Quinolinic acid | −2 | 4 | 1 | 3 | 0.313 |
| 3-Hydroxybutyric acid | −3 | 5 | 1 | 4 | 0.188 |
| L-Phenylalanine | −4 | 4 | 0 | 4 | 0.063 |
| Alpha-D-Glucose | −5 | 7 | 1 | 6 | 0.063 |
| L-Glutamine | −5 | 5 | 0 | 5 | 0.031 |
| N-acetyl glycoprotein | −5 | 7 | 1 | 6 | 0.063 |
| L-Lactic acid | −8 | 8 | 0 | 8 | 0.004 |
| *LysoPC*, lysophosphatidylcholine. | | | | | |

| **Supplementary Table 16. Vote-counting results for blood in non-antidepressant.** | | | | | |
| --- | --- | --- | --- | --- | --- |
| **Metabolites** | **Vote-counting statistic** | **No. of studies that report on the metabolite** | | | ***P* value** |
|  |  | **All** | **Upregulated** | **Downregulated** |  |
| Trimethylamine N-oxide | 22 | 24 | 23 | 1 | <0.001 |
| L-Isoleucine | 18 | 20 | 19 | 1 | <0.001 |
| L-Leucine | 10 | 10 | 10 | 0 | 0.001 |
| Creatine | 9 | 19 | 14 | 5 | 0.032 |
| L-Phenylalanine | 8 | 10 | 9 | 1 | 0.011 |
| Serotonin | 8 | 8 | 8 | 0 | 0.004 |
| L-Valine | 7 | 9 | 8 | 1 | 0.020 |
| L-Tryptophan | 6 | 10 | 8 | 2 | 0.055 |
| Pyruvic acid | 6 | 12 | 9 | 3 | 0.073 |
| Betaine | 5 | 7 | 6 | 1 | 0.063 |
| Indoleacrylic acid | 4 | 4 | 4 | 0 | 0.063 |
| L-Acetylcarnitine | 4 | 4 | 4 | 0 | 0.063 |
| LDL | 4 | 4 | 4 | 0 | 0.063 |
| Hippuric acid | 2 | 4 | 3 | 1 | 0.313 |
| L-Palmitoylcarnitine | 2 | 4 | 3 | 1 | 0.313 |
| L-Proline | 2 | 4 | 3 | 1 | 0.313 |
| L-Tyrosine | 2 | 4 | 3 | 1 | 0.313 |
| Cholic acid | 1 | 7 | 4 | 3 | 0.500 |
| L-Glutamic acid | 1 | 11 | 6 | 5 | 0.500 |
| L-Threonine | 1 | 5 | 3 | 2 | 0.500 |
| Citric acid | 0 | 8 | 4 | 4 | 0.637 |
| L-Methionine | 0 | 6 | 3 | 3 | 0.656 |
| LysoPC(16:0) | 0 | 6 | 3 | 3 | 0.656 |
| Sphingosine 1-phosphate | 0 | 4 | 2 | 2 | 0.688 |
| Succinic acid | 0 | 4 | 2 | 2 | 0.688 |
| Deoxycholic acid | −1 | 5 | 2 | 3 | 0.500 |
| D-Glucose | −1 | 5 | 2 | 3 | 0.500 |
| Lipid | −1 | 5 | 2 | 3 | 0.500 |
| LysoPC(15:0) | −2 | 4 | 1 | 3 | 0.313 |
| Phytosphingosine | −2 | 4 | 1 | 3 | 0.313 |
| Sphinganine | −2 | 8 | 3 | 5 | 0.363 |
| 3-Hydroxybutyric acid | −4 | 10 | 3 | 7 | 0.172 |
| Allantoin | −4 | 4 | 0 | 4 | 0.063 |
| Arachidonic acid | −4 | 10 | 3 | 7 | 0.172 |
| Choline | −4 | 18 | 7 | 11 | 0.240 |
| Oxoglutaric acid | −4 | 4 | 0 | 4 | 0.063 |
| Taurine | −4 | 4 | 0 | 4 | 0.063 |
| Glycine | −6 | 6 | 0 | 6 | 0.016 |
| HDL | −6 | 6 | 0 | 6 | 0.016 |
| myo-Inositol | −6 | 6 | 0 | 6 | 0.016 |
| Phenylacetylglycine | −6 | 6 | 0 | 6 | 0.016 |
| Beta-D-Glucose | −8 | 8 | 0 | 8 | 0.004 |
| L-Alanine | −8 | 12 | 2 | 10 | 0.019 |
| Corticosterone | −10 | 10 | 0 | 10 | 0.001 |
| L-Glutamine | −10 | 10 | 0 | 10 | 0.001 |
| N-acetyl glycoprotein | −13 | 19 | 3 | 16 | 0.002 |
| L-Lactic acid | −15 | 19 | 2 | 17 | <0.001 |
| Alpha-D-Glucose | −20 | 24 | 2 | 22 | <0.001 |
| *LDL*, low-density lipoprotein; *LysoPC*, lysophosphatidylcholine; *HDL*, high-density lipoprotein. | | | | | |

| **Supplementary Table 17. Vote-counting results for urine in antidepressant.** | | | | | |
| --- | --- | --- | --- | --- | --- |
| **Metabolites** | **Vote-counting statistic** | **No. of studies that report on the metabolite** | | | ***P* value** |
|  |  | **All** | **Upregulated** | **Downregulated** |  |
| Citric acid | 3 | 5 | 4 | 1 | 0.188 |
| Hippuric acid | −1 | 5 | 2 | 3 | 0.500 |

| **Supplementary Table 18. Vote-counting results for urine in non-antidepressant.** | | | | | |
| --- | --- | --- | --- | --- | --- |
| **Metabolites** | **Vote-counting statistic** | **No. of studies that report on the metabolite** | | | ***P* value** |
|  |  | **All** | **Upregulated** | **Downregulated** |  |
| Oxoglutaric acid | 9 | 9 | 9 | 0 | 0.002 |
| Acetic acid | 8 | 8 | 8 | 0 | 0.004 |
| Betaine | 6 | 6 | 6 | 0 | 0.016 |
| Creatinine | 6 | 10 | 8 | 2 | 0.055 |
| Phenylacetylglycine | 5 | 5 | 5 | 0 | 0.031 |
| Creatine | 4 | 4 | 4 | 0 | 0.063 |
| Kynurenic acid | 4 | 8 | 6 | 2 | 0.145 |
| Uric acid | 4 | 4 | 4 | 0 | 0.063 |
| Xanthurenic acid | 4 | 6 | 5 | 1 | 0.109 |
| Citric acid | 3 | 17 | 10 | 7 | 0.315 |
| L-Proline | 3 | 5 | 4 | 1 | 0.188 |
| Succinic acid | 3 | 7 | 5 | 2 | 0.227 |
| Allantoin | 2 | 4 | 3 | 1 | 0.313 |
| L-Asparagine | 2 | 4 | 3 | 1 | 0.313 |
| L-Serine | 0 | 4 | 2 | 2 | 0.688 |
| Trimethylamine | 0 | 6 | 3 | 3 | 0.656 |
| L-Tyrosine | −1 | 5 | 2 | 3 | 0.500 |
| Pyruvic acid | −1 | 7 | 3 | 4 | 0.500 |
| Cyclic AMP | −2 | 4 | 1 | 3 | 0.313 |
| Hippuric acid | −2 | 12 | 5 | 7 | 0.387 |
| L-Phenylalanine | −2 | 6 | 2 | 4 | 0.344 |
| Taurine | −2 | 8 | 3 | 5 | 0.363 |
| Glycine | −5 | 5 | 0 | 5 | 0.031 |
| Cortisol | −6 | 6 | 0 | 6 | 0.016 |
| *AMP*, adenosine monophosphate. | | | | | |

| **Supplementary Table 19. Vote-counting results for the liver in non-antidepressant.** | | | | | |
| --- | --- | --- | --- | --- | --- |
| **Metabolites** | **Vote-counting statistic** | **No. of studies that report on the metabolite** | | | ***P* value** |
|  |  | **All** | **Upregulated** | **Downregulated** |  |
| L-Glutamic acid | 4 | 10 | 7 | 3 | 0.172 |
| Glutathione | 4 | 6 | 5 | 1 | 0.109 |
| Ornithine | 4 | 4 | 4 | 0 | 0.063 |
| L-Lysine | 3 | 5 | 4 | 1 | 0.188 |
| Oxoglutaric acid | 2 | 6 | 4 | 2 | 0.344 |
| Glycerol | 0 | 4 | 2 | 2 | 0.688 |
| Taurochenodesoxycholic acid | −3 | 5 | 1 | 4 | 0.188 |
| L-Tryptophan | −4 | 4 | 0 | 4 | 0.063 |
| Alpha-D-Glucose | −5 | 5 | 0 | 5 | 0.031 |
| Arachidonic acid | −5 | 5 | 0 | 5 | 0.031 |
| L-Lactic acid | −6 | 8 | 1 | 7 | 0.035 |
| Beta-D-Glucose | −6 | 6 | 0 | 6 | 0.016 |

| **Supplementary Table 20. Vote-counting results for feces in non-antidepressant.** | | | | | |
| --- | --- | --- | --- | --- | --- |
| **Metabolites** | **Vote-counting statistic** | **No. of studies that report on the metabolite** | | | ***P* value** |
|  |  | **All** | **Upregulated** | **Downregulated** |  |
| Butyric acid | 7 | 9 | 8 | 1 | 0.020 |
| Acetic acid | 7 | 7 | 7 | 0 | 0.008 |
| Propionic acid | 5 | 9 | 7 | 2 | 0.090 |
| Isovaleric acid | 4 | 4 | 4 | 0 | 0.063 |
